# Timing in Conversation

**DOI:** 10.5334/joc.268

**Published:** 2023-04-05

**Authors:** Antje S. Meyer

**Affiliations:** 1 Radboud University Nijmegen and Max Planck Institute for Psycholinguistics and Radboud University, Nijmegen, The Netherlands

**Keywords:** conversation, turn-taking, speech planning

## Abstract

Turn-taking in everyday conversation is fast, with median latencies in corpora of conversational speech often reported to be under 300 ms. This seems like magic, given that experimental research on speech planning has shown that speakers need much more time to plan and produce even the shortest of utterances. This paper reviews how language scientists have combined linguistic analyses of conversations and experimental work to understand the skill of swift turn-taking and proposes a tentative solution to the riddle of fast turn-taking.

This paper concerns the timing of speech planning in conversation. Conversation is important for our everyday lives. We use it to pass the time and bond with strangers, to conduct sales talks and selection interviews, to teach, and to derive medical diagnoses. It is where children acquire language, and, as many experienced during the covid-19 pandemic, it is something people really crave. As it is such a common and socially important type of human behavior it should be of central interest to cognitive and language scientists. Studying conversation is also important for practical reasons. Though it is typically experienced as effortless, conversation can become taxing in persons with speech or language impairments, for instance after a stroke, in persons with hearing loss and non-native speakers of a language. To support such individuals, diagnosis of their difficulties is required, which presupposes a clear view of typical conversation. Finally, conversations occur not only face-to-face, but also in remote contexts (e.g. video conferencing; Boland, Fonsesca, Mermelstein, & Williamson, 2021) and in interactions with “smart” home appliances or chat facilities of service providers. To optimize the conditions for conversation in such contexts, in particular for making them feel natural, a good understanding of typical face-to-face conversation is needed.

For all of these reasons, studying conversation is valuable. In addition, it is essential for assessing the scope of psycholinguistic processing models of speaking and listening. These models are largely based on experimental work carried out in laboratory environments, which differ in many ways from the environments where conversations are typically held (Kandylaki & Bornkessel-Schlesewsky, 2019; Kuhlen, Bogler, Brennan, & Haynes, 2017; Kuhlen & Rahman, 2022; Sjerps, Decuyper, & Meyer, 2019; Verga & Kotz, 2019). For instance, rather than conversing with another person, participants in lab experiments are typically tested individually, and they produce utterances in monologues or respond to recorded utterances. These utterances are often short and similar across many trials (e.g., they may be series of single nouns produced as picture names) and appear without any broader context. An important working assumption in experimental psycholinguistics is that processing principles uncovered in laboratory work also hold in other contexts. This implies, for instance, that the order and timing of processes occurring when a word is retrieved for speaking in the lab or in a conversation are essentially the same. The working assumption is reasonable as participants performing linguistic tasks in the lab likely apply skills they have acquired through everyday language use. Nonetheless it possible that linguistic processes are speeded up or slowed down when they occur in different contexts, or, more importantly, that speakers prefer different processing strategies. Thus, to assess the scope of psycholinguistic theories, it is necessary to determine whether the mechanisms postulated on the basis of laboratory work can also support speaking and listening in natural conversation.

In sum, there are important practical and theoretical reasons for studying conversation. The specific issue addressed in the current paper concerns the speed of conversational turn-taking. Linguists and psycholinguists have often commented on the fluency of natural conversation, the fact that speakers can respond to each other almost instantaneously. The short gaps between turns contrast sharply with the long speech onset latencies for words and sentences in laboratory contexts. This discrepancy gives rise to two questions. First, how can conversational turn-taking be so fast? Second, what does this mean for the validity of theories of speech planning that are tailored to explain the relatively slow speech planning in the lab?

In this paper, I first provide a brief characterization of conversation, and then review and discuss research addressing the timing of turn-taking. The goals of the paper are, first, to illustrate how experimental psycholinguistics and linguistic approaches to conversation can be combined to understand how language is used in natural contexts, and second, to propose and motivate a specific account of rapid turn-taking.

## Key properties of conversation

Conversations occur in many different contexts and vary widely in, for instance, the geographical surroundings where they take place, the demographic properties of the participants, the level of formality, and their content. People can have conversations almost anywhere about anything. Nonetheless, conversations have core properties, which result from rules that the interlocutors spontaneously observe. These core properties and rules have been extensively described and discussed in the sociolinguistic and linguistic literature. Much of this work has been done within the framework of conversation analysis (Sachs, Schegloff, & Jefferson, 1974; Schegloff, 1968; 2007; Schegloff, Jefferson & Sachs, 1977; Schegloff & Sachs, 1973) or was inspired by work in this framework (see Clark, 1996, for a different approach, and Horton, 2017, for a review).

Four key properties of conversations are relevant for the present purposes. First, conversations are social events and involve at least two participants. An individual can only have a monologue. Second, conversations consist of turns. Turns are, broadly speaking, the speakers’ contributions to the conversation. Their length and form are not fixed. They can be single words (for instance, an emphatic “Coffee!”), short phrases (“no milk!”), or longer utterances. In addition, there are backchannels, such as “uhu” or “yeah”, which listeners use to encourage their partners to continue their turns and which are often not classified as turns themselves (e.g., Bangerter & Clark, 2003; Knudsen, Creemers, & Meyer, 2020; Schegloff, 1982; Tolins & Fox Tree, 2014, 2016). Third, successive turns are pragmatically linked, that is, they fit in the context of the conversation. Questions need relevant answers, requests need to be accepted or rejected, stories need relevant comments, and so on. The different types of links between turns in conversation have been extensively discussed in the linguistic and sociolinguistic literature (e.g., Albert & De Ruiter, 2018; Goodwin, 1981; Kendrick & Torreira 2015; Roberts, Torreira, & Levinson, 2015; Sacks, Schegloff, & Jefferson, 1974; Schegloff, 1968, 2000, 2007; Stivers & Rossano, 2010). For the present purposes, it suffices to note that speakers in conversation mostly provide contextually appropriate responses to each other.

The fourth property, which is most central for this paper, is the temporal coordination between turns. Most of the time only one person talks and the speakers’ turns follow each other promptly. Levinson and Torreira (2016, page 6) note that “the system is highly efficient: less than 5% of the speech stream involves two or more simultaneous speakers (the modal overlap is less than 100 ms long), the modal gap between turns is only around 200 ms, and it works with equal efficiency without visual contact”. Support for the claim that turns are tightly coordinated in time comes from corpus analyses. For instance, in a much-cited study Stivers and colleagues (2009) examined the gaps between yes/no questions and the following answers in ten languages and found median gap durations between 0 ms and 300 ms. Similarly, Heldner and Edlund (2010) found median gap durations around 100 ms in corpora of Dutch, English, and Swedish conversational speech. Furthermore, linguistic analyses suggest that gap durations may carry meaning. For example, an unexpectedly long gap may express reluctance to accept a request, which indicates that, as a rule, turns are tightly linked in time (e.g., Barthel & Sauppe, 2019; Bögels, Kendrick, & Levinson, 2015; Kendrick & Torreira, 2015). Relatedly, Templeton, Chang, Reynolds, Cone LeBeaumont, and Wheatley (2022) found that faster response times in informal conversations were correlated with stronger feelings of social connection and with more enjoyment of the conversations, perhaps because fast responding is experienced as indicative of paying attention and understanding each other.

The tight coordination of turns in content and timing shows that speakers generally succeed in planning and producing a turn very shortly after the end of the preceding turn. This is remarkable because utterance planning is not instantaneous but requires substantial amounts of time. For instance, in lab experiments participants typically need 600 ms to 800 ms to name a line drawing of a common object (e.g., Indefrey, 2011; Indefrey & Levelt, 2004), and preparing a simple sentence can easily require a second or more (e.g., Ferreira, 1991; Konopka, 2019). These long planning times are not surprising given the complexity of the conceptual and linguistic encoding processes to be performed. For a short phrase, the encoding processes include deciding which concepts to talk about, selecting appropriate words to express them, generating the grammatical structure of the utterance, and retrieving the phonological, phonetic and articulatory codes (e.g., Roelofs & Ferreira, 2019). Even though these processes may overlap in time, the entire encoding process is complex and requires processing time. One might think that answering questions or making thoughtful comments in a conversation would require more time, not less, than performing the simple laboratory tasks.

## Levinson and Torreira’s model of turn-taking

The gaps between turns appear to be mysteriously short only as long as one assumes that comprehension and production of turns occur strictly in sequence; i.e. that a person first listens to all of the interlocutor’s turn and then begins to plan a response. The mystery is solved if listening and response planning are allowed to overlap in time, i.e. if speakers begin to plan a turn before the end of the partner’s turn. For many turn sequences, this is plausible. For instance, in a café a customer might not need to hear much more than “What can …?” to know that the barista is ready to take the order and to respond accordingly.

Levinson and Torreira (2015) proposed a working model of conversational turn-taking that captures the idea that listening and speech planning overlap in conversation. They assume that in conversation each participant’s production system and their comprehension system are active in parallel. The listener’s task is to identify the partner’s speech act and gist. The speech act is the type of action accomplished in the turn; common speech acts are requests, questions, and statements (e.g., Austin, 1962; Searle, 1979). The gist is, broadly speaking, what the utterance is about. Both speech act and gist constrain the appropriate answer. For instance, a listener hearing a tourist ask “Do you know how to get to the train station?” must understand that a simple “Yes, I do.” is not the answer the tourist is hoping for. As soon as the listener has sufficient evidence about the speech act and gist of the partner’s turn, they can begin to plan their response. This can often be well before the end of the turn, as illustrated in the above utterance “What can…?” uttered by a barista. When there is sufficient evidence that the turn will soon end, the listener – now next speaker – can launch the prepared utterance. This means that the articulators are prepared and the utterance is initiated. Thus, short gaps between turns arise because listeners take certain risks in basing their response preparation on parts of the partner’s turn, and in launching them when they anticipate, rather than hear, the end of the turn.

## Listeners predict speaker meaning and ends of turns

Levinson and Torreira’s model is important for the language sciences because it bridges between descriptive linguistic work on conversation and lab-based psycholinguistic work. This is because it explains the coordination between turns in time and content by reference to specific cognitive processes: early recognition of gist and speech act, prediction of ends of turns, and early response preparation. The model can be evaluated by assessing, first, whether these processes indeed take place and, second, whether they lead to short gaps between turns. Conducting such a research program is not straightforward because most experimental paradigms require participants to carry out specific tasks at specific times and therefore cannot be used while speakers are engaged in spontaneous conversation. However, one can ask whether the central claims of the model are consistent with laboratory findings and current theories of speech processing and planning. This question is discussed in the current and the next section of this paper.

Two key assumptions concern listening in conversation. The first one is that listeners can grasp the partner’s meaning before the end of their utterance. This assumption is consistent with a strong body of evidence showing that speech processing is highly incremental and opportunistic, with all available evidence immediately being used to infer the meaning and to predict upcoming parts of the utterance (Dahan & Ferreira, 2019; Huettig, 2015; Huettig, Audring, & Jackendoff, 2022; Kuperberg & Jaeger, 2015). In addition, there are studies showing specifically that listeners can rapidly infer the speech act of utterances (e.g., Bögels & Torreira, 2015; Gisladottir, Bögels, & Levinson, 2018; Gisladottir, Chwilla, & Levinson, 2015; Nota, Trujillo, & Holler, 2021; Tomasello, Grisoni, Boux, Sammerler, & Pulvermuller, 2022).

The second claim is that listeners predict ends of turns and launch prepared responses in anticipation rather than in response to them. This claim is consistent with the strong evidence for prediction during language processing already mentioned above and with specific evidence concerning listeners’ ability to predict ends of turns. For instance, Corps, Gambi, and Pickering (2020) showed that participants in a laboratory study used both the global speech rate of yes/no questions they had to answer and the duration of the final word of the question to predict the end of the question and time their answer accordingly. In addition, there is a substantial literature specifically concerning the prediction of ends of turns. Linguistic analyses have shown that there are many cues that can foreshadow the ends of turns (for a useful listening, see Rühlemann & Gries, 2020). These cues include, for instance, tag questions, such as “Isn’t it?”, phonetic cues, such as pitch drops and turn-final lengthening of words, and gestural cues. Laboratory studies where participants were asked to press a button as soon as they thought a turn had ended have shown that listeners are sensitive to such cues and can use them to anticipate ends of turns, rather than respond to them (e.g., de Ruiter, Mitterer, & Enfield, 2006; Magyari, Bastiaansen, de Ruiter, & Levinson, 2014; Magyari & de Ruiter, 2012). Other laboratory studies have demonstrated that listeners can use semantic information and the discourse context to predict ends of turns (e.g., Bögels & Torreira, 2021; Corps, Pickering, & Gambi, 2019; Riest, Jorschick, & de Ruiter, 2015). However, in conversational speech, speakers use such cues quite inconsistently (e.g., Gravano & Hirschberg, 2011), and little is known about the cues listeners actually attend to in predicting ends of turns in conversation (for further discussion see Barthel, Meyer, & Levinson, 2017; Bögels, 2020; Brehm & Meyer, 2021; Corps, Crossley, Gambi, & Pickering, 2018).

## Utterances are planned early and launched later

The third claim of Levinson and Torreira’s model concerns the timing of speech planning: Listeners, aka next speakers, begin to plan their utterances as soon as they have enough information to do so. This claim implies that listening and speech planning often occur at the same time. It is this head-start in speech planning relative to the ends of turns that, according to this account, leads to the short gaps between turns.

But can speakers prepare utterances while listening? And does such early preparation for speaking indeed contribute to short gaps between turns? This is not self-evident, as one might expect listening and speech planning to interfere with each other. However, several experiments have shown that speech planning during listening is indeed possible and that it facilitates fast responding. The first relevant experiment was carried out by Bögels, Magyari, and Levinson (2015). The participants heard quiz questions, such as “Which character, also called 007, appeared in the famous movies?” or “Which character from the famous movies is also called 007?”, which differed in the position of the cue to the answer (“007” in the example) in the sentence. If participants begin to plan their response as soon as all relevant information is available, they should respond sooner when the cue appears early than when it appears late in the question. This prediction was borne out, with the average response latency being shorter by about 300 ms in the early-cue than in the late-cue condition. Moreover, EEG recordings during the task suggested that planning during listening progressed to the level of phonological form retrieval (see also Barthel & Levinson, 2020; Bögels, 2020; Bögels, Casillas, & Levinson, 2018; for discussion of the neurophysiological evidence see Jongman, Piai, & Meyer, 2020).

Studies using related paradigms found compatible pattern of results (e.g., Barthel, Sauppe, Levinson, & Meyer, 2016; Magyari, de Ruiter, & Levinson, 2017; Meyer, Alday, Decuyper, & Knudsen, 2018). For instance, Corps, Crossley, Gambi, and Pickering (2018) asked participants about personal experiences and opinions using questions that had highly predictable endings (e.g., “Are dogs your favourite animal?”) or less predictable endings (e.g., “Have you visited the city of Paris?”). The questions with predictable endings, which allowed for early response planning, were answered faster than the questions with less predictable endings. In sum, all of these studies showed that participants can begin to plan answers during ongoing questions and thereby reduce their response latencies.

It is, however, worth noting that upcoming speakers do not necessarily begin to plan utterances as early as possible. For instance, in a study by Sjerps and Meyer (2015), participants first heard a description of a quadruple of objects (“The spoon moves above the house and the dog moves below the key”), and then had to describe another quadruple in the same way. Importantly, they could see both quadruples from the beginning of the trial and all utterances had the same structure and involved lexical items of similar difficulty. Therefore, the participants could estimate quite well how long the interlocutor’s utterance would be and how long they would need to prepare the first part of their own utterance. Their eye movements showed that they usually only started to look at their own quadruple and began to plan the utterance when the interlocutor was about to name the last of the four objects. This study shows that, contrary Levinson and Torreira’s proposal, upcoming speakers do not necessarily start planning utterances as soon as the relevant information is available. When the interlocutor is likely to produce a lengthy utterance (e.g., when a parent “lectures” a teenager about bad behaviour), listeners may postpone response planning and so reduce the mental load arising from keeping a planned utterance in working memory.

The fourth claim of Levinson and Torreira’s model is the distinction between response planning and launching: Speakers begin to prepare a response to their partner as soon as possible, but only launch it shortly before the anticipated end of the partner’s turn. This proposal is consistent with a large body of experimental work using delayed naming tasks, which has shown that speakers can indeed generate speech plans internally, retain them in working memory, and produce them upon presentation of a response cue (for recent discussions see Kawamoto, Liu, & Kello, 2015; Krause & Kawamoto, 2020; Piai, Roelofs, Rommers, Dahlstaett, & Maris, 2015; Romani, Silverstein, Ramoo, & Olson, 2022). The latencies to produce prepared utterances are much shorter than those observed for utterances not planned ahead of time. In fact, utterance onset latencies as short as 200 ms after the offset of a verbal cue can only be obtained for utterances that are fully planned and merely have to be launched. This was already demonstrated 150 years ago by Donders (1868), who measured the verbal response speed to verbal prompts (see Roelofs, 2018, for discussion and a partial replication of the historic study). Donders showed that participants could respond with latencies around 400 ms to the onset of a syllable (e.g., “ki”), if there was only a single known response option, namely repeating the stimulus. As the syllables were about 200 ms long, the gap between stimulus offset and response was about 200 ms.

The distinction between early utterance planning and timely launching is crucial for the explanation of short gap durations in conversation. It offers a straightforward explanation for the observation that in many laboratory experiments, participants were, compared with the gap durations in conversation, remarkably slow to begin to speak, even when early response preparation was possible. To illustrate, in the early-cue condition of the quiz study by Bögels and colleagues, participants responded with an average latency of 650 ms, which is more than twice the median gap durations of 200 ms or 300 ms reported for conversational corpora. In similar studies, Bögels, Casillas, and Levinson (2018) observed an average response time of 498 ms for the fastest condition, and Barthel, Sauppe, Levinson, and Meyer (2016) observed an average response time of 749 ms for the fastest condition. A simple account of the long latencies in these studies is that speakers began to prepare their utterances as early as possible, but did not manage to complete their preparation before the end of the question. Hence, more processing than just launching the utterance had to be done after the end of the question, leading to relatively long response times.

This account is consistent with the observation that in some studies much shorter response times were seen. For instance, in the predictable condition of the study by Corps and colleagues (2018) response latencies were just above 200 ms. Apparently, participants could begin to prepare early enough and complete their response preparation before the end of the question. The same was true for a study by Meyer, Alday, Decuyper, and Knudsen (2018), where participants answered yes/no questions about objects on their screen, and for a study by Brehm and Meyer (2021), where participants produced picture names after ample preparation time.

In short, when speakers have sufficient preparation time before a “go” signal, latencies around 200 ms can be observed in the lab. The implication is that in conversation, where short gaps predominate, speakers usually have enough time to prepare their response during the partner’s turn. This point is taken up below after a brief discussion of the coordination of speaking and listening.

## Concurrent speech planning and listening interfere with each other

The model proposed by Levinson and Torreira (2015) implies that speakers begin to plan their utterances while listening to their interlocutor, and, as discussed, numerous studies have now confirmed that speech planning can indeed occur at the same time as listening. These results lead to the question how speakers perform this form of linguistic dual-tasking, for instance, whether they conduct both tasks in parallel or switch rapidly between listening and speaking. Surprisingly little work has been conducted on this issue. One clear result, which has direct implications for understanding conversation, is that concurrent speech input hampers speech planning, making it slower and more error-prone than speech planning performed on its own. Incoming speech affects speech planning in two ways: by forcing the speaker to distribute attention across speech planning and comprehension, and by creating cross-talk between similar representations.

Turning first to the division of attention, numerous studies have shown that both listening and speaking require some attention. Clear demonstrations of the attention demands of these processes come from studies where participants either talk themselves or listen to speech while performing a concurrent motor task that demands attention (e.g., Almor, 2008; Boiteau, Malone, Peters, & Almor, 2014; Fargier & Laganaro, 2016; Ferreira & Pashler, 2002). Under such dual-task conditions performance in the linguistic or/and motor task is typically worse than when each task is performed by itself. This pattern shows that speaking and listening require attention: If some attention is needed for the motor task, performance in the concurrent linguistic task suffers. Roelofs and colleagues (e.g., Roelofs, 2021; Roelofs & Piai, 2011) developed and thoroughly tested a detailed theory of the involvement of attention in speaking.

Similarly, many studies have shown that speech comprehension requires attention. This holds in particular for higher-level processes, such as syntactic integration and reanalysis and drawing inferences (for recent discussion and reviews see Cohen, Salony, Pallier, & Dehaene, 2021; Hubbard & Federmeier, 2021; Jacquemot & Bachaud-Levi, 2021; Wehbe et al., 2021).

Another reason why speech planning is hampered by concurrent speech input is that planning and processing speech are related cognitive activities, as both require access to the words and grammatical rules of the language. Interference effects have been shown in numerous picture-word-interference experiments, where participants were asked to name pictures while hearing or seeing written distractor words, which they should ignore (e.g., Schriefers, Meyer, & Levelt, 1990). Compared to silence or noise baselines or to speech that participants cannot understand (e.g. Chinese speech for native speakers of Dutch, He, Meyer, Creemers, & Brehm, 2021), the presentation of distractor words in the participants’ own language slows down picture naming. Moreover, with suitable timing of the distractors, semantically related distractors (e.g., “cat” for the picture of a dog) slow down naming more than unrelated ones (e.g. “fork” for the picture of a dog; see Burki, Elbuy, Madec, & Vasishth, 2020, for a review). A standard account of these findings relies on the assumption of a shared mental lexicon for word production and comprehension. The spoken distractor word and the concept invoked by the picture both activate entries in the mental lexicon. Related entries (e.g. cat and dog) activate each other and compete for selection. This competition must be resolved, which requires processing resources and slows down naming (e.g., Levelt, Roelofs, & Meyer, 1999; Roelofs, 1992; for an alternative account see Mahon, Costa, Peterson, Varga, & Caramazza, 2007). Incoming speech draws upon a speaker’s processing capacity, even when they do not aim to listen to the input but try to ignore it.

In sum, speech planning and processing incoming speech compete for attention, and speech input can interfere with the selection of words for production and slow down planning. Hence, planning utterances while listening to speech is bound to be slower and more error-prone than planning in the absence of concurrent speech (see also Barthel & Sauppe, 2019; Fairs, Bögels, & Meyer, 2018). This explains, among other things, why participants in the quiz study by Bögels and colleagues and in studies using related paradigms benefitted from early cues to the answer, but still responded well after the offset of the question.

## Alignment may support fast responding

As just shown, it is not difficult to explain why the participants in laboratory experiments often needed several hundred milliseconds to initiate responses to simple questions. However, the need to divide attention between listening and speech planning and interference from the spoken input should arise in conversation as well, and so the question remains how speakers in conversation nonetheless manage to respond to each other with the observed short gaps between their turns.

A number of proposals have been made about ways in which speakers in conversation could facilitate each other’s speech planning. The most prominent among them is mutual alignment, highlighted in seminal work by Garrod and Pickering (2004; Pickering & Garrod, 2004). Briefly, the basic idea is that in conversation speakers align on all levels of representation, for instance by using the same word (e.g., “shoe” or “loafer”) to refer to an object under discussion, and by repeating syntactic structures. In other words, speakers prime each other, and perhaps themselves, at different levels of representation, and this priming facilitates mutual understanding and speech planning.

The rich literature on alignment cannot be reviewed here (for discussion see Ivanova, Horton, Swets, Kleinman, & Ferreira, 2020; Rasenberg, Ozyurek, Bogels, & Dingemanse, 2022). There is no doubt that speech planning can be primed. For instance, there is strong evidence from many laboratory studies demonstrating lexical repetition priming, with words being retrieved faster and/or more accurately when they have been recently heard or produced than when this is not the case (e.g., Bartolozzi, Jongman, & Meyer, 2021; Francis, Gurrola, & Martinez, 2022; Tsuboi, Francis, & Jameson, 2021). There is also laboratory evidence for syntactic priming, with speakers’ likelihood of using a given structure increasing after recent experience of that structure. This holds in particular for relatively infrequent structures (e.g., Ferreira & Bock, 2006; Jacobs, Cho, & Watson, 2019; Pickering & Ferreira, 2008; Tooley, 2022). There is also some evidence that syntactic priming may speed up utterance formulation (Segaert, Wheeldon, & Hagoort, 2016; Hardy, Wheeldon, & Segaert, 2020), though in general syntactic priming affects the choice of structures more than the speed of producing them. How strongly each of these priming mechanisms supports speech planning in conversation remains to be determined.

## Incremental planning and control of utterance form yield fast but often disfluent responses

A second potentially important reason why response planning in conversation can be fast is that speakers can choose what they say and how much of their utterance they plan before beginning to speak. By contrast, in laboratory experiments, participants are typically asked to produce well-formed utterances of specific formats (e.g. sentences such as “The woman gives the man a cup”) and to avoid hesitations and repairs. Even under those circumstances, participants often choose not to plan the entire utterance but only a first chunk, often corresponding to one or two words, before beginning to speak. This strategy can lead to disfluencies or pauses after the first chunk (e.g., Brown-Schmidt & Konopka, 2015; Konopka, 2019; Lee, Brown-Schmidt, & Watson, 2013; Roelofs & Ferreira, 2019; Papafragou & Grigoroglou, 2019). In conversation, speakers can also plan utterances incrementally, and, for instance, only plan the first two words of their turn. Moreover, they can choose how to start, for instance, by beginning with an easy-to-plan particle, such as “Well…”. Such incremental planning allows speakers to take up their turn quickly, but, as in laboratory experiments, it may lead to disfluencies later in the utterance. In fact, conversational speech is riddled with disfluencies, i.e. silent and filled pause, repetitions, errors and repairs, suggesting that speakers often make use of highly incremental planning strategies and prioritize speed – fast responding to the partner – over well-formedness and fluency (e.g., Arnold, Tanenhaus, Altmann, & Fagnano, 2004; Clark & Fox Tree, 2002; Crible, 2019; Crible & Pascual, 2020; Fox Tree & Clark, 1997). Why speakers set their priorities in this way needs to be further studied. In some contexts, for instance, in multi-party conversations, speakers must respond fast to seize the floor, but short gaps between turns are also observed in casual dyadic conversations, where there is little competition for the floor (e.g., Holler et al., 2021). In such contexts swift responding appears to contribute to a feeling of social connection between the interlocutors (e.g., Templeton et al., 2022). The main point to note here is that flexibility in word choice and in the span of advance planning may facilitate speedy responding in conversation.

## Do speakers have enough planning time?

Regardless of the mechanisms and strategies that may support fast responding in conversation, speakers always need some time to hear and understand at least the beginning of the partner’s utterance (e.g., the first word of the turn, as in “Dinner ready?”), to decide what to say, to retrieve an appropriate word or phrase as an answer (“Not yet.”), and to launch it. As discussed above, speakers need to have a complete speech plan for the beginning of their utterance to respond to a partner within a few hundred milliseconds. Given laboratory results concerning the time needed for speech planning, it is unlikely that a complete speech plan, even for a short utterance, can be created in much less than a second. This means that turns have to be at least about 800 ms long to receive responses with gaps of 200 ms.

How long are turns in conversation? In the published literature, there is surprisingly little information about turn durations. There are many phonetic studies of conversational speech where information about utterance durations must have been gathered but is not reported, presumably because this information was not of interest to the researchers. Levinson (2016) suggests an average turn duration of about two seconds, which would give speakers sufficient time to respond with a short gap to information provided early in the turn. Based on analyses of an English corpus of telephone conversations (Calhoun et al., 2010) Levinson and Torreira (2015) report an average turn duration of 1680 ms and a median of 1227 ms.

To add to this literature, Corps, Knudsen, and Meyer (2022) set out to examine the distribution of turns of different length in corpora of conversational speech in American English, Dutch, and German. Here we discuss the German corpus, which they analysed most extensively (see also Knudsen, Creemers, & Meyer, 2020). The analyses confirmed that the speakers’ utterances seamlessly followed each other, with mean and median gap durations close to zero. The average duration of the utterances was two seconds, corresponding to seven words. Thus, on average, upcoming speakers, had enough time to plan their utterances. However, the distributions of the utterances were highly skewed, with short utterances being far more common than long ones. The median was one second, or three words, and the mode (the most common utterance length) was just one word. Regardless of how much time speech planning takes, whether it is half a second or a second, many utterances were shorter than the shortest plausible estimate of planning time.

This result is puzzling. How can the gaps between the speakers’ utterances be so short when the current speaker’s utterance is too short to allow the next speaker to prepare a response? Further analyses of the corpus showed that many of the utterances that were automatically labelled as turns were not complete turns, but only parts of turns. This situation most commonly arose when the speakers talked at the same time, as is illustrated in (1). Referring to a bar discussed earlier, Speaker B says “Ok, da war aber halt nichts los.” (“Ok, but nothing happened there.”), and the other speaker simultaneously says “Da beim Chinesen nebendran, gell? (“There next to the Chinese <restaurant>, right?”). In the transcript, the two parallel utterances are aligned word-by-word and rendered, incorrectly, as an exchange of one- or two-word turns.

**Table:** 

(1)	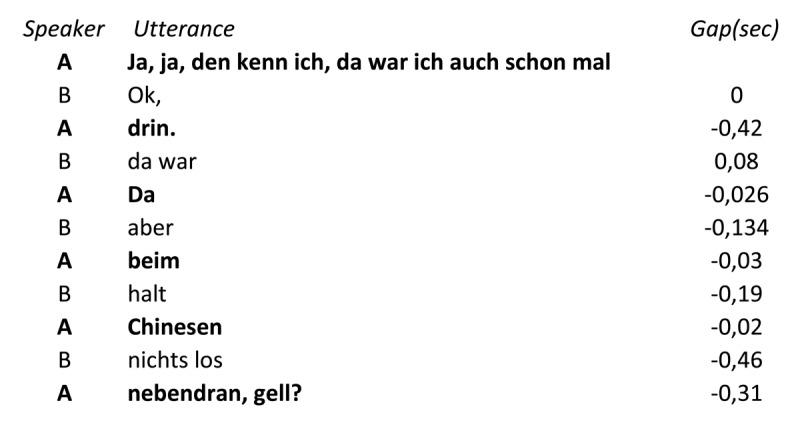

To assess how often this situation arose, Corps and colleagues categorized each automatically defined segment as a self-continuation or a different type of segment. Self-continuations were defined purely in syntactic and lexical terms, e.g. when a segment missed a verb phrase that was provided in the next segment by the same speaker, or by the use of pronouns referring to a preceding segment. The use of these stringent criteria allowed for transparent and replicable coding of the segments. Corps and colleagues found that 24% of the segments were self-continuations. For the purpose of determining the length of turns self-continuations should be combined with the preceding segment by the same speaker. When this was done, the average turn duration rose to 6.0 seconds, and the median to 3.4 seconds. The gap between turns remained close to zero, with a mean of –.09 seconds and a median of –0.02 seconds. Thus, in contrast to the initial impression based on the automatic parsing of the utterances, these results suggest that the speakers in this conversation usually did have enough time to prepare a turn while their partner was talking. It is important to stress that the above turn durations only concern the relatively small German corpus analyzed by Corps and colleagues. Further work is needed to obtain a better estimate of the proportions of self-continuations and the durations of turns in informal conversation.

The analyses carried out by Corps and colleagues also showed that the speakers often did not use all of the time afforded by the partner’s utterance to plan their own turn and launch it shortly before the end of the partner’s turn. Instead, they often began to speak much earlier. As noted already, 24% of the segments stemmed from episodes of parallel talk, and 9% of the turns were fully embedded in longer turns, i.e. began after and ended before the end of a partner’s turn. Why do speakers talk at the same time? In the linguistic literature parallel talk has often been linked to premature turn-taking (e.g., Drew, 2009; Schegloff, 2000): A speaker picks up on part of the partner’s utterance and begins to respond while the partner is still talking. This holds for the turns in (1), where Speaker A confirms, quite elaborately, that they know the bar, while Speaker B already talks about the fact that said bar is rather boring. In other words, it is not the case that speakers in parallel talk do not respond to the partner’s utterance content. They do respond, but their turns strongly overlap in time. In the corpus discussed here, this happened often; whether this is generally the case in casual conversation remains to be seen. In the phonetic and linguistic literature, the existence of parallel talk has been widely acknowledged (e.g., Jefferson, 1986, 2004; Kurtić & Gorisch, 2018), but no estimates of its prevalence in conversation seems to be available.

Parallel talk is similar to the use of backchannels, which are utterances such as “uhu” or “ehem”. In the German corpus analyzed by Corps and colleagues, 23% of the segments were backchannels. They are often not considered to be turns themselves, but as encouragement to the current speaker to continue their narrative or elaborate on what they said before (e.g., Tolin & Fox Tree, 2014, 2016). Importantly, as backchannels introduce no new propositional content, the current speaker does not have to respond to such content, and so the question how they manage to rapidly grasp the other speaker’s meaning and respond to it does not arise. As in parallel talk, the current speaker just continues their turn.

## Summary and conclusions

The goals of this paper were, first, to illustrate how experimental psycholinguistics and linguistic approaches to language can be combined to understand how language is used in conversation, and second, to propose and motivate a specific account of rapid turn-taking. To turn to the first goal, Levinson and Torreira’s (2015) model is an excellent starting point for interdisciplinary studies of conversation because it is based on insights from linguistic theory and corpus analyses, but is also a processing model with claims about speaking and listening in conversation and the coordination of these processes. As was discussed above, the model can be evaluated with respect to its consistency with existing psycholinguistic theories and findings, and it can be tested in new empirical work. For instance, the quiz study by Bögels and colleagues (2015) and several later studies on utterance planning during listening were specifically designed to test the assumption that speakers already begin to plan their utterance during the partner’s turn. This turned out to be the case. These studies led not only to novel insights about conversation, but also contributed to psycholinguistic theories, for instance, to theories about the capacity demands of speaking and listening (e.g., Barthel & Sauppe, 2019; Sjerps & Meyer, 2015). Laboratory research had shown that speakers need to fully plan their utterances to be able to start speaking within 200 ms after the end of another speaker’s utterance. This finding triggered new corpus analyses by Corps and colleagues (2022) aiming to investigate whether turns in conversation are generally long enough to allow for complete utterance preparation. The analyses showed, first, that many automatically determined speech segments were not turns, and, second, that speakers often talked in parallel rather than immediately responding to each other. In this line of research, linguistic analyses and experimental psycholinguistic work were tightly intertwined and led to new insights into the way interlocutors achieve timely turn-taking. Of course, others have pointed out the need to combine linguistic and psycholinguistic approaches to conversation (e.g., De Ruiter & Albert, 2017). Here the aim was to highlight this important point again and to illustrate in some detail how corpus analyses and experimental work can be brought together to study a specific research question.

The second goal was to address the question how speakers manage to respond to each other almost instantaneously. We offer two complimentary answers. The first answer was already proposed by Levinson and Torreira (2015). Gaps between turns can be short because listeners can often quickly grasp the gist and speech act of the partner’s utterance, prepare a response, and launch it when the end of the partner’s turn is imminent. As explained above, this proposal is broadly consistent with current theories and findings from lab-based psycholinguistics, which have shown, for instance, that sentence processing is highly incremental and predictive, such that speakers can indeed rapidly grasp the content and speech act of turns and predict ends of turns, and with the evidence that speakers can prepare utterances while listening to another person’s speech.

The second answer is that speakers in conversation often do not respond directly, segment-by-segment, to the content just expressed by their partner. Instead one person talks, while the other provides backchannels, or the speakers develop their turns in parallel. In parallel talk, speakers engage in linguistic dual-tasking but the need to respond rapidly and appropriately to the partner’s utterance does not arise. Parallel talk may occur when a speaker responds to the content expressed early in the partner’s turn, perhaps anticipating that the turn would end sooner than it actually did.

The two answers are related. Both imply that listeners quickly grasp the meaning of the partner’s turn and begin to formulate a response. “Neat” sequential turn-taking, with one speaker responding close to the end of the other’s turn, occurs when the second speaker estimates correctly when the partner’s turn will end and times their fully prepared utterance to coincide closely with that event. As discussed above, achieving such tight coordination of turns is no mean feat and requires accurate prediction of turn ends, in parallel with response planning and timely launching of the prepared utterance, as described in Levinson and Torreira’s model. In parallel talk, the upcoming speaker also plans a response during the interlocutor’s turn, but times it to begin well before the end of the partner’s turn, either misjudging how long the partner will continue talking or simply not taking this into account. Talking during concurrent speech input requires a speaker to divide their attention between listening and speech planning, and the selection of words for speaking may be hampered because of interference from the spoken words. This may lead to hesitant speech featuring silences and filled pauses. Speakers might find it difficult to predict ends of turns in hesitant speech, which may lead to further parallel talk. This is how long stretches of parallel talk may arise.

Further empirical and theoretical work is needed to flesh out and test this proposal and, more generally, understand how participants in conversation coordinate their utterances in time and content. The model proposed by Levinson and Torreira (2015) has stimulated much research and its key assumptions are consistent with existing laboratory work and/or have been confirmed in targeted investigations. However, for many aspects of conversational turn-taking precise functional models are still missing. For instance, it is still far from clear how interlocutors manage to simultaneously process their partner’s utterance and prepare and often even produce their response, and which cues in the partner’s utterance they use to predict their end of turn and the right time to launch their utterance.

In addition, very little is known about the way speech comprehension and speech planning processes interface with motivational processes and social cognition, which likely strongly shape both the content and the timing of conversations. Here, an important open question is why casual conversation adheres to tight time constraints in the first place. Why do people prefer to respond swiftly to each other, even though this affects the fluency and well-formedness of their utterance? And why do they talk in parallel even though this must be effortful and may affect mutual understanding? As mentioned earlier, swift responding has been linked to enjoyment and a feeling of social connection, i.e. of being heard and understood. This is plausible, but one might wonder why a feeling of social connection is linked to fast, rather than slow (and thoughtful) responding. It has also been proposed that conversation is a form of joint action, which requires well-coordinated responses (e.g., Garrod & Pickering, 2009). The feeling of acting together in a conversation may only arise when each partner speaks at the expected response time. This also seems plausible, but again one might ask why joint conversational action needs to be fast rather than well-measured. An interesting speculation was offered by Levinson (2016), who proposed that during the evolution of human language, turn-taking initially served the exchange of very short utterances, which could readily be generated with short latencies. Later, languages became more complex, but the turn-taking system remained geared towards short swift exchanges.

To gain a better understanding of these issues and the cognitive processes underlying conversation, corpus analyses must be combined with experimental work. In the corpus work, researchers need to use or generate richly annotated corpora, where turns and sub-turn units are tagged, and where gaps between turns can be distinguished from other inter-speaker gaps. As illustrated above, transcripts based solely on phonetic information will often not suffice to identify the beginnings and ends of turns. Suitable corpora have been generated in different labs, for instance by Kendrick and Holler (2017), Roberts, Torreira, and Levinson (2015), and Skantze (2021). However, recent evidence has highlighted the importance of visual information for turn-taking (e.g., Holler & Levinson 2019; Holler, Kendrick & Levinson, 2018). Thus, for in-depth studies of the timing of conversation, multi-modal corpora are required. Moreover, it would be highly desirable to use corpora covering a broad range of conversations, so that the variability in the timing of conversations across settings can be determined. To illustrate, one might expect less parallel talk in formal settings, such as job interviews, than in conversations among friends. If the view proposed here is correct, the gaps between turns should be longer in more formal contexts than in casual conversation.

Richly annotated multi-modal corpora provide descriptions of the interlocutors’ behaviour. They reveal what the speakers say, which gestures they make, and when they do so. They also reveal how the speakers’ utterances are related in time and content. By their very nature, spontaneous conversations offer researchers no control over the participants’ behavior, and so analyses of conversational speech are not sufficient for testing processing theories of speaking and listening in conversation. Therefore, corpus analyses need to go hand-in-hand with experimental work. Here, the challenge is to design experimental paradigms that allow for stringent control of the variables of interest in settings that optimally approximate natural conversation.
